# Beneficial Effect of Alkaloids From *Sophora alopecuroides* L. on CUMS-Induced Depression Model Mice *via* Modulating Gut Microbiota

**DOI:** 10.3389/fcimb.2021.665159

**Published:** 2021-04-19

**Authors:** Ming Zhang, Aoqiang Li, Qifang Yang, Jingyi Li, Lihua Wang, Xiuxian Liu, Yanxin Huang, Lei Liu

**Affiliations:** ^1^National Engineering Laboratory for Druggable Gene and Protein Screening, Northeast Normal University, Changchun, China; ^2^Jilin Provincial Key Laboratory of Animal Resource Conservation and Utilization, Northeast Normal University, Changchun, China; ^3^School of Rehabilitation Science, Shanghai University of Traditional Chinese Medicine, Shanghai, China

**Keywords:** depression, gut microbiota, function prediction, *Sophora alopecuroides* L., alkaloids, CUMS

## Abstract

It was recently shown that the gut microbiota of both depression patients and depression model animals is significantly altered, suggesting that gut microbes are closely related to depression. Here, we investigated the effects of *Sophora alopecuroides* L.-derived alkaloids on the gut microbiota of mice with depression-like behaviors. We first established a mouse model of depression *via* chronic unpredictable mild stress (CUMS) and detected changes in depression-like behaviors and depression-related indicators. Simultaneously, 16S rRNA sequencing was performed to investigate gut microbiota changes. *Sophora alopecuroides* L.-derived alkaloids improved depression-like behaviors and depression-related indicators in mice. The alkaloids decreased the gut microbiota diversity of CUMS mice and depleted intestinal differentially abundant “harmful” microbiota genera. Spearman analysis showed that there is a certain correlation between the differential microbiota (*Lactobacillus*, *Helicobacter*, *Oscillospira*, *Odoribacter*, *Mucispirillum*, *Ruminococcus*), depression-like behaviors, and depression-related indicators. Combined with the predictive analysis of gut microbiota function, these results indicate that alkaloids improve depression in mice through modulating gut microbiota.

## Introduction

Depression is a common mental and emotional disorder that has become a major contributor to the global burden of disease (Yamagata et al., 2017). According to statistics from the World Health Organization in 2015, the incidence rate of depression exceeded 18%, and the disability rate was higher than that for any other disease (Winter et al., 2018). Depression is characterized by disorders of the neurotransmitter, neuroimmunity, neuroendocrine and metabolic systems; however, the pathogenesis of depression is still unclear (Zhang et al., 2015; Ma et al., 2016; Song et al., 2019).

Studies have shown that there is a complex network relationship between the brain and the gut microbiota (Bienenstock and Collins, 2010). The gut microbiota can have an important impact on the host’s stress response, depression, and cognitive function through the gut-brain axis, which is closely related to depression (Mayer, 2011). Clinical studies have found that the composition of the gut microbiota in patients with depression is altered compared with that of healthy people, and the diversity and abundance of the gut microbiota of patients have decreased significantly (Jiang et al., 2015; Aizawa et al., 2016; Liu et al., 2016). Gut microbiota disorders increase the susceptibility to depression (Frei et al., 2012; Bercik and Collins, 2014; Oriach et al., 2016; Köhler et al., 2017; Owen and Corfe, 2017). Improvement in the gut microbiota can relieve depression-like symptoms, while probiotic treatment can significantly reduce patients’ depression and anxiety symptoms (Akkasheh et al., 2016; Pirbaglou et al., 2016; Abildgaard et al., 2017; Gibson et al., 2017). Traditional antidepressants, such as trans-cyclopropylamine and imipramine, have been found to have antibacterial effects while improving depression (Macedo et al., 2017). A new therapy consisting of a selective serotonin reuptake inhibitor (SSRI) combined with probiotics to improve intestinal microbes has a good antidepressant effect. These studies have confirmed that the treatment of depression can be achieved by regulation of the gut-brain axis (Schnorr and Bachner, 2016; Bambling et al., 2017).

*Sophora alopecuroides* L. (*S. alopecuroides*) is widely distributed in Xinjiang. Its main ingredient is alkaloids, which have antitumor, antibacterial, anti-inflammatory, and other biological activities (Zhang et al., 2017). Previous studies have shown that alkaloids from other plants have good antidepressant effects (Coetzee et al., 2016; Hamid et al., 2017; Jia et al., 2017; Wu et al., 2018; Arias et al., 2019; Chen et al., 2019; Khan et al., 2019), while the effect of alkaloids from *S. alopecuroides* on depression and gut microbes has not been investigated. In the current study, we used chronic unpredictable mild stress (CUMS) to establish a depression mouse model and assess the changes in depression-like behaviors and depression-related indicators to evaluate the beneficial effect of alkaloids on CUMS depression-like mice. We also used amplicon sequencing to study how the composition and structure of the gut microbial community in depression-like mice changed between different treatment conditions. The correlation between gut microbes and depression-related indicators was further analyzed. In this study, we aimed to provide experimental evidence for further research on how alkaloids from *S. alopecuroides* can improve depression in mice by regulating gut microbiota.

## Materials and Methods

### Preparation and Analysis of Total Alkaloids

The seeds of *S. alopecuroides* were collected in October 2016 from Xinjiang Altai, China. The plant species was identified by the Xinjiang Food and Drug Administration. A voucher specimen (sa-201610-003) was deposited in the National Engineering Laboratory for Druggable Gene and Protein Screening, Northeast Normal University, Changchun. The specific process of extracting total alkaloids was as follows: briefly, the air-dried samples (10 kg) were ground into 60 mesh powder, ultrasonically extracted with 75% ethanol (5 × 2 h) at 30°C, and evaporated using a rotary evaporator (SENCO Technology Co., Ltd, China) to remove the ethanol. The ethanol extract was dissolved in 0.5% dilute hydrochloric acid solution, passed through a strong acid cation exchange resin column for full exchange, and then washed with water to neutrality. The resin was removed and dried and then wetted with 14% ammonia water. Finally, it was extracted with 95% ethanol, and the total alkaloids content was obtained after rotary evaporation. Identification of total alkaloids was performed by HPLC-MS (Prominence LC-20A, Japan) by using an SPD 20AV HPLC DAD coupled to an API 2000 quadrupole-MS (SCIEX, USA). Chromatographic separation was performed on a ZORBAX SB-C18 (150×2.1 mm, 5 μm) column (Agilent Technologies, USA). The flow rate was 1.0 mL/min, and mass spectra were recorded in positive ionization.

### Animals and Groups

**Figure 1:**
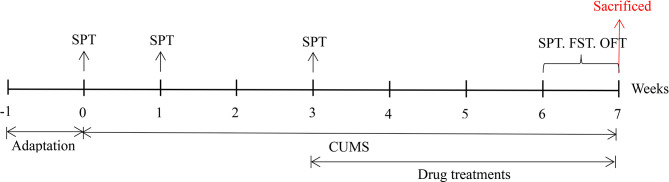
The specific experimental design and implementation. SPT, sucrose preference test; FST, forced swimming test; OFT, open field test.

Male ICR mice (20-25 g) were purchased from Liaoning Changsheng Biotechnology Co., Ltd. (Changchun, China), housed with a constant temperature (21 ± 1°C) and humidity (52 ± 2%) on a 12 h day/night cycle in a laboratory room, and given a standard diet and water ad libitum. After the mice had adapted to the new environment for one week, their body weight and sucrose preference were measured to remove outliers. The mice were divided into four groups (n = 10): the control group (Con, not exposed to CUMS), CUMS group, CUMS + imipramine group (CUMS + IMI) and CUMS + alkaloids group (CUMS + ALK). All procedures were performed in accordance with the guidelines for the National Institutes of Health guide for the care of laboratory animals and were approved by the Experimental Animal Care and Use Committee at Northeast Normal University. The specific experimental design is shown in **Figure 1**.

### Chronic Unpredictable Mild Stress

The CUMS procedure was performed according to the literature with slight modifications (Huang et al., 2018; Liu et al., 2019). Mice were isolated and subjected to two different stressors every day, and the same stressors were not scheduled for two consecutive days to ensure that the animals could not predict the occurrence of the stress. These stressors were applied for seven weeks, and the details are shown in **Table 1**.

**Table 1:** The schedule of CUMS stressors.

Weeks	Days						
	Mon.	Tue.	Wed.	Thur.	Fri.	Sat.	Sun.
Week 1	E+C	D+G	N	L+B	M+J	A+F	K+H
Week 2	K+G	D+C	N	E+A	H+B	L+M	F+J
Week 3	E+K	M+J	N	A+I	F+C	L+G	H+N
Week 4	H+J	E+D	N	F+L	K+C	A+I	M+G
Week 5	M+G	H+C	N	A+L	F+B	E+J	K+D
Week 6	D+C	E+G	N	I+B	K+F	A+J	H+M
Week 7	E+G	D+J	N	A+L	F+K	B+C	M+H

A-food deprivation for 12 h; B-water deprivation for 12 h; C-lights on at night for 12 h; D-empty cage for 12 h; E-wet bedding for 12 h; F-confinement in a tube for 2 h; G-traffic noise (70-90 dB) for 2 h; H-tail suspension for 0.5 h; I-cage tilting for 12 h (45°); J-exposure to a stroboscope for 12 h; K-foreign body stimulation for 2 h; L-crowding for 12 h (ten mice within one cage); M-level shaking for 15 min; N-food and water deprivation for 24 h.

### Treatments

The drug treatments began from the 4th week during the CUMS procedure and lasted four weeks. The four groups of mice were treated as follows: Con (0.9% saline), CUMS (0.9% saline), CUMS + IMI (30 mg/kg imipramine), and CUMS + ALK (30 mg/kg alkaloids). Except for the intraperitoneal injection of the imipramine group, all other groups underwent intragastric administration. Imipramine hydrochloride is a listed drug for treating depression and was used as a positive control in this experiment. Imipramine hydrochloride and alkaloids were dissolved in 0.9% saline at a stock concentration of 3 mg/ml. Drug treatments were performed at the same time every day (9:00 a.m.-11:00 a.m.).

### Sucrose Preference Test

A sucrose preference test (SPT) procedure was performed as described in the literature, with slight modifications (Liu et al., 2018). The SPT was performed at four time points: before CUMS (adaptation period, Monday to Thursday), before drug treatment (Monday to Thursday in the first and third weeks), and before the animals were sacrificed (Monday to Thursday in week 7). Mice were housed in single cages during the test. At the start of the test, mice were trained for 48 h to adapt to a 1% sucrose solution (w/v) and avoid the bottle place preference: two bottles of 1% sucrose solution were placed in each cage for 24 h, one of the bottles of 1% sucrose solution was replaced with a bottle of pure water for 12 h, and the positions of the two bottles were then exchanged for 12 h. Finally, the mice were deprived of food and water for 24 h. The SPT was then performed, with two bottles randomly placed, one bottle containing a sucrose solution and the other bottle containing pure water. The mice drank freely for 2 h, and the bottles were weighed before and after placement. The sucrose preference was calculated as a percentage of the consumed sucrose solution relative to the total amount of liquid consumed.

### Forced Swimming Test

The forced swimming test (FST) has been used to evaluate the antidepressant effect of drugs. The shorter the immobility time is, the stronger the antidepressant effect of the drug (Sun et al., 2018). The FST was performed before the animals were sacrificed (Friday of week 7). The experimental device used for forced swimming was a cylindrical plastic container (25 cm height × 10 cm diameters) containing 25 ± 1 °C water, and the depth of the water was 20 cm. Each mouse was individually placed in the above device in a quiet environment and forced to swim for 6 min, which was recorded through a digital camera. After swimming, the mice were dried and quickly returned to their home cage. The device was washed with clean water before each FST. Throughout the entire swimming test, the first 2 min were the pre-swimming phase, and the remaining 4 min were used to calculate the immobility time. The time during which the mice gave up struggling to float on the water surface or only slightly swing their limbs to prevent immersion in the water was counted as immobility time.

### Open Field Test

The open field test (OFT) has been widely used to study the behaviors associated with neuropsychological changes in experimental animals, and it can be used to evaluate the effects of antidepressant treatment (Shyong et al., 2017). The OFT was performed before the animals were sacrificed (Sunday of week 7). The device used was a black open box (30 cm×30 cm×30 cm), and the bottom of the apparatus was divided into 16 equally sized squares. Each mouse was placed in the center of the device in a quiet room for 6 min, and the first 1 min was the adaptation period. We used a digital camera to record the activities of the next 5 min, including the numbers of crossings in the horizontal direction (entry onto the square with all 4 limbs was counted as 1 time), numbers of rearings (the mouse’s forelimbs were counted once when they left the bottom surface), and the numbers of modifications (modifying their heads or mouth with their forelimbs was counted as 1). After each test, the excrement in the box was cleaned, and the box was wiped with an alcohol pad to ensure that there was no smell or dirt from the test mouse.

### Sample Collection

After the mice were decapitated, blood was collected immediately with a blood collection tube and then centrifuged at 3000 rpm for 10 min at 4°C to separate serum and blood cells. The hippocampus and prefrontal cortex were rapidly separated on ice plates, frozen immediately in liquid nitrogen and then stored in a refrigerator at −80°C. The cecum was rapidly removed for collection of intestinal contents (a set of sterile forceps and scissors was used for each mouse to avoid cross-contamination) and frozen in liquid nitrogen until DNA extraction.

### Enzyme-Linked Immunosorbent Assay

The brain-derived neurotrophic factor (BDNF) protein concentration in the hippocampus and prefrontal cortex and the concentrations of serotonin (5-HT), norepinephrine (NE) and dopamine (DA) in serum were measured using the corresponding commercial enzyme-linked immunosorbent assay (ELISA) kits (Enzyme-linked Biotechnology Co., Ltd., Shanghai, China) following the manufacturer’s instructions.

### DNA Extraction

The DNA of intestinal content samples was extracted using a DNeasy PowerWater Kit (QIAGEN, Inc., Netherlands) following the manufacturer’s instructions and stored at -20°C for further analysis. A NanoDrop ND-1000 spectrophotometer (Thermo Fisher Scientific, Waltham, MA, USA) and agarose gel electrophoresis were used to measure the quantity and quality of DNA samples.

### 16S rRNA Gene Amplicon Sequencing

The V3–V4 region of bacterial 16S rRNA genes was amplified using the forward primer 338F (5′-ACTCCTACGGGAGGCAGCA-3′) and reverse primer 806R (5′-GGACTACHVGGGTWTCTAAT-3′) (Xiao et al., 2019). The genes were amplified under an appropriate amplification system and parameters. Agencourt AMPure Beads (Beckman Coulter, Indianapolis, IN) and a PicoGreen dsDNA Assay kit (Invitrogen, Carlsbad, CA, USA) were used to purify and quantify the PCR amplicons, respectively. Equimolar concentrations of the purified amplicons were pooled and sequenced on the Illumina NovaSeq-PE250 platform.

Sequence processing was performed using Quantitative Insights Into Microbial Ecology version 2 (QIIME2, 2019.4 release) (Bolyen et al., 2018). Briefly, we imported the demultiplexed paired-end fastq files into the QIIME2 pipeline. Cutadapt was used to remove primer sequences and discard the unmatched reads (Martin, 2011). Quality filtering, denoising and chimera removal were performed using the DADA2 plugin in QIIME2, with default settings (Callahan et al., 2016). After denoising, amplicon sequence variant (ASV) feature sequences and tables were generated, and singleton ASVs were removed. To generate taxonomy tables, ASV feature sequences were taxonomically assigned using the feature-classifier classify-sklearn plugin, with a naïve Bayes classifier trained on the Greengenes database 13.8 (DeSantis et al, 2006). Furthermore, to standardize sampling effort across samples, the ASV table was rarefied by the diversity alpha-rarefaction plugin according to 90% of the minimum sample frequency.

### Statistical Analysis

All biological results were confirmed as the mean ± standard deviation (SD) of at least three independent experiments. SPSS (Abacus Concepts, Berkeley, CA, USA) and Prism 8.0 (GraphPad, San Diego, CA, USA) software were used. A t test was used to analyze the significant differences between treatments.

Alpha diversity measures (the Chao1 richness index and Shannon diversity index) were produced by the QIIME2 diversity alpha plugin to analyze the alpha diversity level for different treatment groups. The nonparametric Kruskal-Wallis test was used to compare the differences in alpha diversity between different groups since normal distributions of the data or homogeneity of variances were rejected according to the Shapiro and Bartlett test of the package *stats*, respectively. We then used the false discovery rate (FDR) correction to calculate pair comparisons between group levels (*post hoc* analyses) for multiple testing. Bray-Curtis distances were used to calculate the beta diversity and were visualized with principal coordinates analysis (PCoA) in the R package *vegan*. A nonparametric analysis of variance (PERMANOVA) based on 999 permutations was used to test for differences in community structure among groups, and *post hoc* pairwise testing (pairwise differences between groups) was assessed with the function “*pairwise.adonis*” from the package *pairwiseAdonis*. Homogeneity of dispersion among sample groups was also assessed using the *betadisper* function (Arbizu and PairwiseAdonis, 2019; Oksanen, 2019). We also performed analysis of similarity (ANOSIM) to determine whether the difference between groups was greater than the difference within the groups. To identify the ASVs most responsible for differences among groups, linear discriminant analysis (LDA) effect size (LEfSe) was performed with the default recommended settings (Segata et al., 2011). Taxon abundances at the phylum and genus levels were statistically compared between groups by Metastats (White et al., 2009) and visualized as column plots. A taxonomic tree was produced to display the composition of all taxonomic levels. In addition, to verify the correlation between the gut microbiota genera and depressive-like behaviors and depression-related indicators, the relative abundance of the top 10 ASVs was selected for redundancy analysis (RDA) and the Spearman correlation test. The RDA was generated by R software (v4.0.3, package vegan). Spearman correlations were calculated and plotted by R (v4.0.3, package corrplot). The metabolic potential of the gut microbiota was predicted by Phylogenetic Investigation of Communities by Reconstruction of Unobserved States (PICRUSt2) analysis using the KEGG Pathway Database.

## Results

### Alkaloids

Total alkaloids from the seeds of *S. alopecuroides* were analyzed by HPLC-MS (positive ionization). The results showed the presence of six main alkaloids, which were identified as oxymatrine, oxysophoridine, sophoridine, sophocarpine, matrine, and lupanine (Liu et al., 2011; Wang et al., 2012) (**Supplementary Figure 1** and **Supplementary Table 1**).

### Effect of Alkaloids on Depression-Like Behaviors in Mice

**Figure 2:**
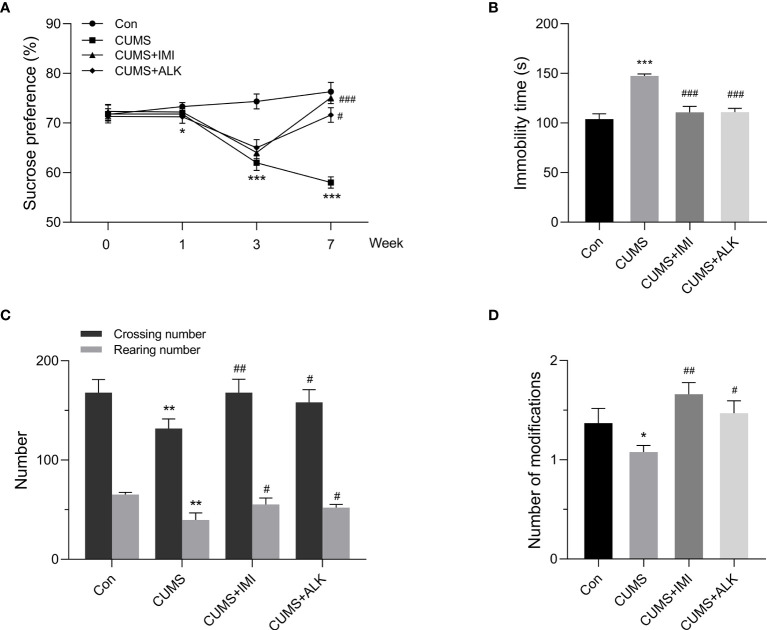
Effect of alkaloids on depression-like behaviors. **(A)** Sucrose preference in the SPT. **(B)** Immobility time in the FST. **(C)** The number of crossings and rearings in the OFT. **(D)** The number of modifications in the OFT. Data are presented as the mean ± SD. *P < 0.05, **P < 0.01, ***P < 0.001 versus the control group (Con); ^#^*P* < 0.05, ^##^*P* < 0.01, ^###^*P* < 0.001 versus the CUMS group.

The SPT, FST and OFT were used to evaluate the effect of alkaloids on depression-like behaviors in CUMS mice. As shown in **Figure 2A**, the SPT was performed four times (at 0, 1, 3, and 7 weeks). Compared with that of the control group mice, the sucrose preference rate of mice under CUMS did not change significantly in the first week. However, a significant difference occurred by the third week (*P* < 0.001), indicating that the depression mouse model may have been successfully established. After 4 weeks of drug treatment, the mice were tested with the SPT on the 7th week. Compared with that of the CUMS group, the sucrose preference rate of the alkaloids (*P* < 0.05) and imipramine (*P* < 0.001) treatment groups was significantly increased. At the same time, the FST and OFT behavioral tests were also carried out in the 7th week. Compared with CUMS alone, alkaloids and imipramine significantly reduced the immobility time in the FST (*P* < 0.001, **Figure 2B**), and the beneficial effects of alkaloids and imipramine were almost the same. Alkaloids also increased the numbers of crossings (*P* < 0.05, **Figure 2C**), rearings (*P* < 0.05) and modifications in the OFT (*P* < 0.05, **Figure 2D**). These results suggest that alkaloids have a significant beneficial effect on the depression-like behaviors of CUMS mice.

### Effect of Alkaloids on the Relative Contents of Depression-Related Indicators in Mice

**Figure 3:**
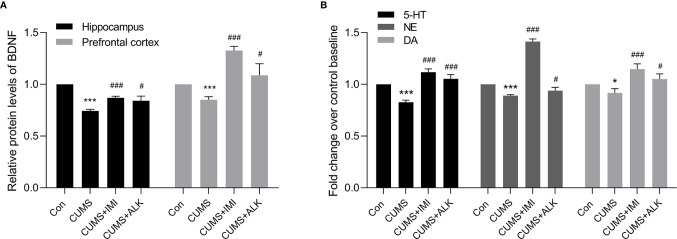
The relative levels of BDNF, 5-HT, NE, and DA. **(A)** The relative protein levels of BDNF in the hippocampus and prefrontal cortex. **(B)** The relative monoamine neurotransmitter (5-HT, NE, and DA) levels in serum. Data are presented as the mean ± SD. *P < 0.05, ***P < 0.001 versus the control group (Con); ^#^*P* < 0.05, ^##^*P* < 0.01, ^###^*P* < 0.001 versus the CUMS group.

Depression is often accompanied by a decrease in the relative content of BDNF and monoamine neurotransmitters (Nemeroff, 2007; Garcia et al., 2008; Racagni and Popoli, 2010; Zhou et al., 2014; Nagy et al., 2020). As shown in **Figure 3**, compared with the control condition, CUMS significantly reduced the relative concentration of the BDNF protein in the hippocampus and prefrontal cortex (*P* < 0.001, **Figure 3A**) and the relative concentrations of 5-HT (*P* < 0.001), NE (*P* < 0.001), and DA (*P* < 0.05) in serum (**Figure 3B**). Moreover, compared with those of the CUMS group, the relative levels of BDNF in the prefrontal cortex and hippocampus and of the monoamine neurotransmitters (5-HT, NE, and DA) in serum were significantly increased after imipramine treatment (*P* < 0.001, **Figures 3A, B**). Alkaloids also increased the levels of BDNF in the hippocampus and prefrontal cortex of CUMS mice (*P* < 0.05, **Figure 3A**) and increased 5-HT (*P* < 0.001), NE (*P* < 0.05), and DA (*P* < 0.05) levels in serum (**Figure 3B**). These results further indicate that alkaloids can improve CUMS-induced depression in mice.

### Microbiota Structure Changes in CUMS Mice Under Alkaloids Treatments

**Figure 4:**
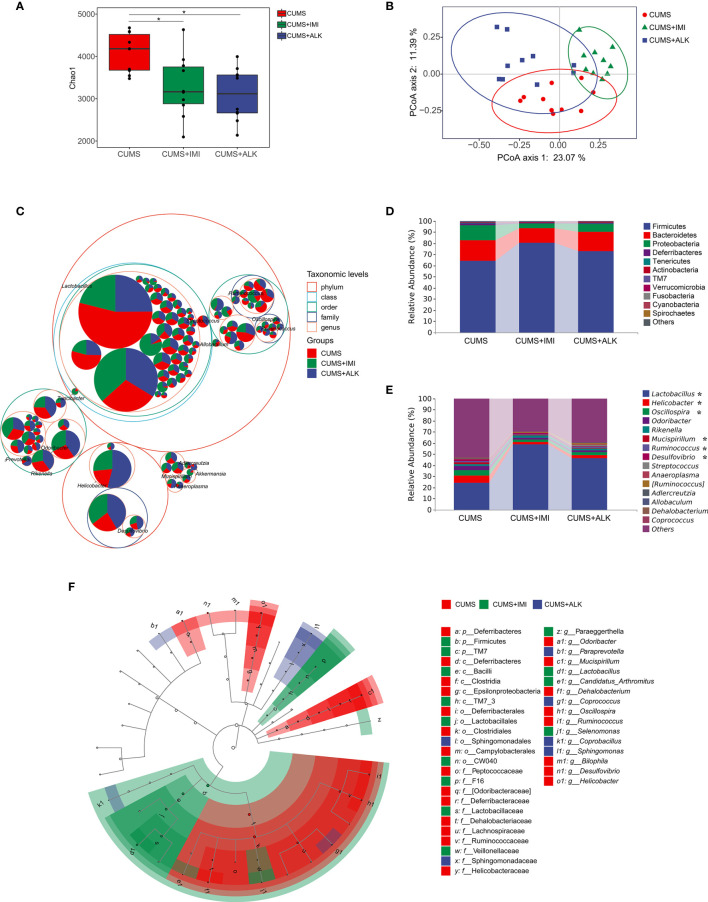
Diversity variation and differential abundance in the gut microbiota composition of mice in different treatment groups. **(A)** The Chao1 index of different treatment groups. Data are presented as the mean ± SE. Mann-Whitney U test results are shown at the top of each paired comparison. *P < 0.05 versus the CUMS group. **(B)** PCoA based on the Bray-Curtis distance for gut microbiota (PCo1 vs. PCo2). Ellipses indicate 95% confidence intervals (CIs). **(C)** Taxonomic tree in packed circles. The largest circles represent the phylum level, and the inner circles represent class, order, family, and genus. The pie chart shows the proportion of each ASV in the different treatment groups. **(D)** Stacked bar chart of the most abundant phyla for CUMS, CUMS+IMI and CUMS+ALK mice. **(E)** Stacked bar chart of the mean relative abundances of bacterial taxa at the genus level for CUMS, CUMS+IMI and CUMS+ALK mice. Asterisks represent microbial taxa with significant differences. **(F)** Cladogram of ASVs with LDA scores greater than 2 based on LEfSe. Different colors represent different groups. Letters represent the position of ASVs in the cladogram.

To explore the changes in the gut microbiome of CUMS mice under alkaloids treatment, we conducted an in-depth analysis of 16S rRNA sequencing. The alpha diversity analysis of intestinal content samples revealed differences within the composition of the microbial community of each group. As shown in **Figure 4**, compared with that of the CUMS group, the Chao1 index of the imipramine and alkaloids treatment groups decreased, but the diversity values between the two groups were not significantly different (**Figure 4A**, CUMS-CUMS+IMI: Padj < 0.05; CUMS-CUMS+ALK: Padj < 0.05; CUMS+IMI-CUMS+ALK: Padj = 0.58). Similar results were obtained for Shannon diversity (**Supplementary Figure 2**, CUMS-CUMS+IMI: Padj < 0.01; CUMS-CUMS+ALK: Padj < 0.001; CUMS+IMI-CUMS+ALK: Padj = 0.58). It is suggested that our drug treatment reduces the diversity of the gut microbiota of CUMS mice, and the effects of alkaloids and imipramine exhibited the same trend.

PCoA results showed that there was a significant clustering difference among the different treatment groups (PERMANOVA: CUMS-CUMS+IMI: F = 3.90, *Padj* = 0.003; CUMS-CUMS+ALK: F = 2.84, *Padj* = 0.006; CUMS+IMI-CUMS+ALK: F = 6.14, *Padj* = 0.003; **Figure 4B**). Furthermore, ANOSIM also confirmed shorter distances between intragroup samples than between intergroup samples (R = 0.506, *P* = 0.001, **Supplementary Figure 3**). In addition, there was no difference in the dispersion of variances among the different treatment groups (*betadisper*: F _(2,27)_ = 3.25, *P* = 0.054). The results showed that there were differences in microbes between different treatment groups, and the difference between the groups was greater than the difference within the groups.

Across all samples, the dominant taxa in the mouse gut microbiota were from three main phyla: Firmicutes, Bacteroidetes, and Proteobacteria (**Figures 4C, D**). After treatment with imipramine and alkaloids, compared with that of the CUMS group, the relative abundance of Firmicutes increased, while the relative abundance of Bacteroidetes and Proteobacteria decreased. However, although the most abundant taxa were similar in different treatment groups at the genus level, the relative abundance of some taxa was different. As shown in **Figure 4E**, compared with that in the CUMS group, the relative abundance of *Lactobacillus* significantly increased in the imipramine and alkaloids groups (CUMS-CUMS+IMI: *Padj* < 0.001; CUMS-CUMS+ALK: *Padj* < 0.05), while the relative abundance of *Oscillospira* significantly decreased (CUMS-CUMS+IMI: *Padj* < 0.01; CUMS-CUMS+ALK: *Padj* < 0.05). Moreover, the relative abundances of *Helicobacter* (CUMS-CUMS+IMI: *Padj* < 0.05)*, Mucispirillum* (CUMS-CUMS+IMI: *Padj* < 0.05) and *Ruminococcus* (CUMS-CUMS+IMI: *Padj* < 0.05) in the imipramine group were significantly reduced, and the relative abundance of *Desulfovibrio* was significantly reduced in the alkaloids group (CUMS-CUMS+ALK: *Padj* < 0.05). In general, at the phylum and genus level, the relative abundance of microbiota between different treatment groups has a certain trend of change.

LEfSe was performed to identify ASVs driving the differences in beta diversity. A total of 41 ASVs were determined to explain the difference in the gut microbiota among different treatment groups (LDA > 2, *P* < 0.05). The results showed that the dominant species in the CUMS group at the genus level were *Odoribacter*, *Mucispirillum*, *Dehalobacterium*, *Oscillospira*, *Ruminococcus*, *Bilophila*, *Desulfovibrio*, and *Helicobacter*. The dominant species at the genus level in the imipramine treatment group were *Lactobacillus*, *Candidatus_Arthromitus*, *Paraeggerthella*, and *Selenomonas*. The dominant species at the genus level in the alkaloids treatment group were *Paraprevotella*, *Coprococcus*, *Coprobacillus*, and *Sphingomonas* (**Figure 4F**). Additionally, the relative abundance levels of each of the abovementioned microbiota genera were assessed in the different treatment groups (the top 50 ASVs at the genus level were selected) (**Supplementary Figure 4**).

### Depression-Like Behaviors and Depression-Related Indicators Correlate With Gut Microbes

**Figure 5:**
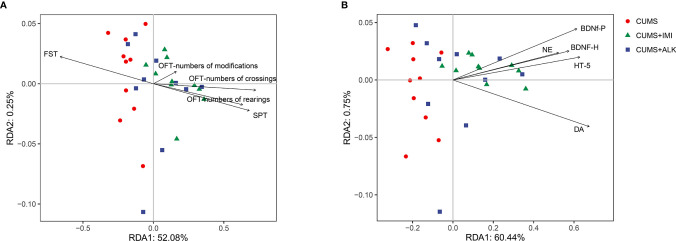
Redundancy analysis (RDA) between gut microbiota, depression-like behaviors, and depression-related indicators. **(A)** RDA was performed to assess the contributions of the depression-like behaviors to the gut microbiota between different treatment groups. **(B)** RDA was performed to assess the contributions of the depression-related indicators to the gut microbiota between different treatment groups.

We performed RDA to study the effects of depression-like behaviors and depression-related indicators on changes in the microbiota genera. The RDA results showed that depression-like behaviors and depression-related indicators influenced the change in the gut microbiota based on the top 10 ASVs at the genus level (**Figure 5**). Among them, SPT (*P* < 0.001), OFT-numbers of crossings (*P* < 0.05), OFT-numbers of rearings (*P* < 0.05), BDNF-h (*P* < 0.001), BDNF-p (BDNF in the prefrontal cortex) (*P* < 0.01), 5-HT (*P* < 0.05), and DA (*P* < 0.01) had significant effects on the gut microbiota. In addition, OFT (numbers of crossings) had the greatest effect on the community structure, accounting for 21.8% of the variation, followed by DA (21.0%), BDNF-h (14.7%), 5-HT (11.1%), OFT-numbers of rearings (11.1%), SPT (10.9%), BDNF-p (9.4%) and DA (10.1%) (**Supplementary Table 2**).

**Figure 6:**
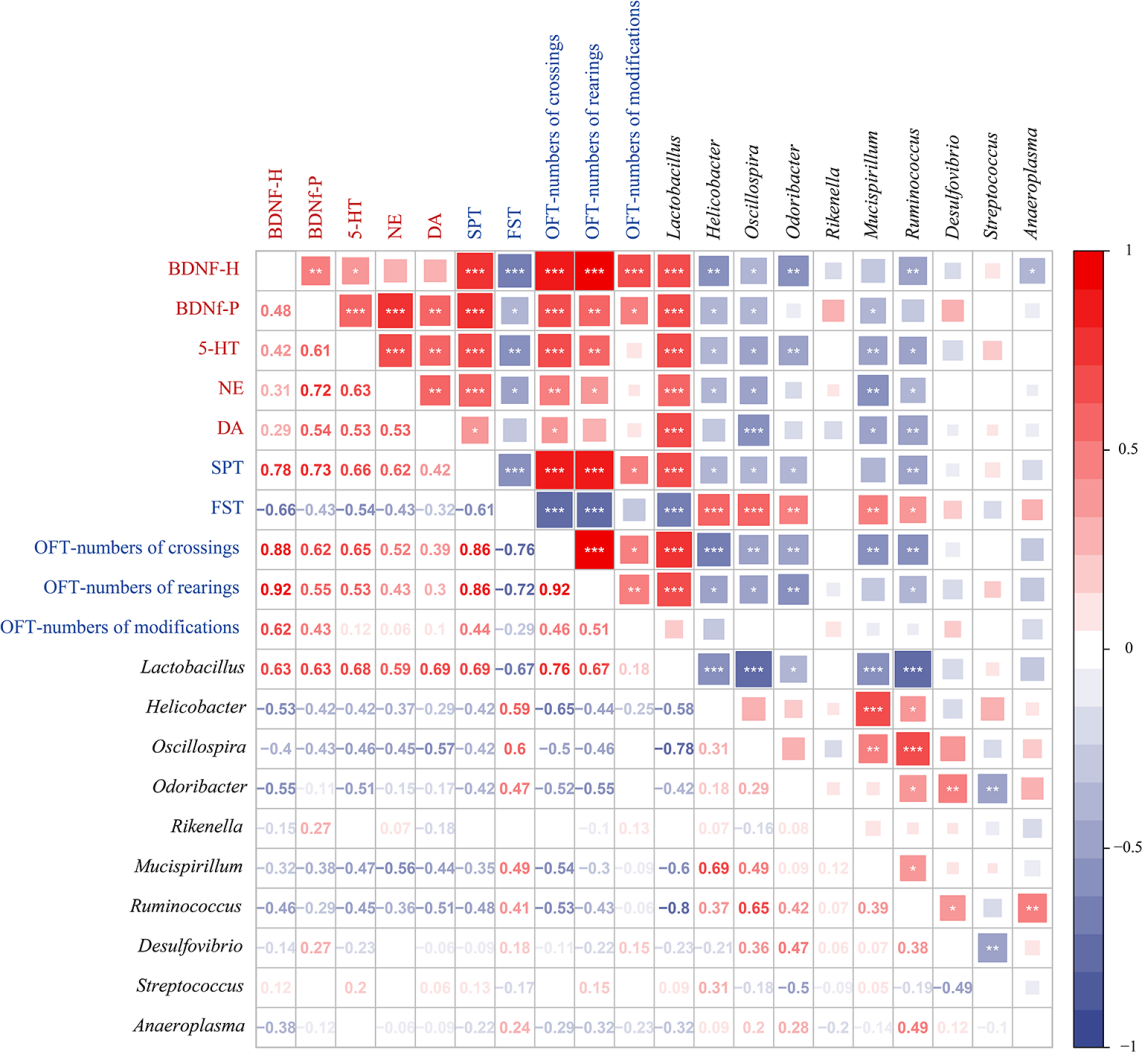
Spearman correlations between gut microbiota, depression-like behaviors, and depression-related indicators. Spearman’s rank correlation coefficient among 5 depression-related indicators, 5 depression-like behaviors, and 10 gut microbiota that differed significantly in abundance between different treatment groups. Axis label: red, depression-related indicators; blue, depression-like behaviors; black, gut microbiota. Numbers on the lower left area: value of correlation coefficient; symbols on the upper right area: results of correlation and significance test, *P < 0.05, **P < 0.01, ***P < 0.001.

To further investigate the correlations among the gut microbiota genera, depression-like behaviors, and depression-related indicators, we analyzed the Spearman correlations (**Figure 6**). We observed that there are 7 genera (*Lactobacillus*, *Helicobacter*, *Oscillospira*, *Odoribacter*, *Mucispirillum*, *Ruminococcus*, and *Desulfovibrio*) among the top 10 genera in relative abundance and differences in LEfSe analysis. Among them, *Lactobacillus*, *Helicobacter*, *Oscillospira*, *Odoribacter*, *Mucispirillum*, and *Ruminococcus* were associated with depression-like behaviors and depression-related indicators. The genera *Helicobacter*, *Oscillospira*, *Odoribacter*, *Mucispirillum*, and *Ruminococcus* were positively correlated with each other and with immobility time in the FST, which was negatively correlated with most of the other depression-like behaviors and depression-related indicators. However, *Lactobacillus* was negatively correlated with immobility time in the FST and with *Helicobacter*, *Oscillospira*, *Odoribacter*, *Mucispirillum*, and *Ruminococcus*. *Lactobacillus* was positively correlated with depression-related indicators, SPT, OFT-numbers of crossings, and OFT-numbers of rearings. These results led us to conclude that changes in the gut microbiota are associated with depression-like behaviors, and depression-related indicators.

### Prediction of Gut Microbiota Function

**Figure 7:**
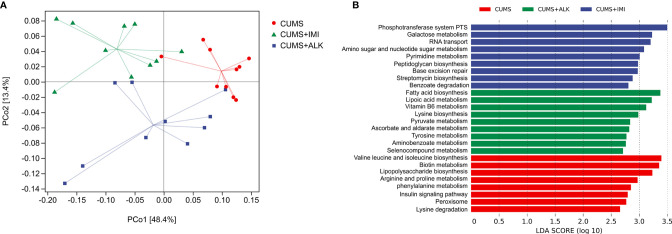
Differences in functional units and metabolic pathways among different treatment groups. **(A)** Principal component analysis of functional units in different treatment groups. PCoA based on Bray-Curtis distance for gut microbiota (PCo1 vs. PCo2). Ellipses indicate 95% confidence intervals (CIs). **(B)** Differential metabolic pathways between different treatment groups, a histogram of linear discriminant analysis (LDA) scores calculated by LEfSe analysis.

To further clarify the correlation between changes in gut microbiota and metabolic pathways, we evaluated the differences in the function of gut microbiota in the CUMS group, imipramine group, and alkaloid group. PICRUSt 2 was used to predict gene content, and classify gene families through the KEGG database. The PCoA results of the functional unit show that there were differences between the groups (**Figure 7A**). The metabolic pathway statistics chart shows that its pathways mainly concentrated in the metabolism, followed by genetic information processing, cellular processes, environmental information processing, organic systems, and human diseases (**Supplementary Figure 5**). The results of LEfSe analysis showed that the differential pathways in the CUMS group were valine leucine and isoleucine biosynthesis, biotin metabolism, lipopolysaccharide biosynthesis, arginine and proline metabolism, phenylalanine metabolism, insulin signaling pathway, peroxisome, and lysine degradation; the differential pathways of the imipramine group were phosphotransferase system PTS, galactose metabolism, RNA transport, amino sugar and nucleotide sugar metabolism, pyrimidine metabolism, peptidoglycan biosynthesis, base excision repair, streptomycin biosynthesis, and benzoate degradation; the differential pathways of the alkaloids group were fatty acid biosynthesis, lipoic acid metabolism, vitamin B6 metabolism, lysine biosynthesis, pyruvate metabolism, ascorbate and aldarate metabolism, tyrosine metabolism, aminobenzoate metabolism, and selenocompound metabolism (**Figure 7B**). These results imply that the gut microbiota directly participates in the metabolic process of the host, which may further affect the changes in depression-related indicators and depression-like behaviors.

## Discussion

Studies have shown that CUMS is a reliable method for establishing depression models (Chen et al., 2015; Davis et al., 2016; Yu et al., 2017). It exposes experimental animals to a variety of unpredictable mild environmental stimuli within a period of time, thereby effectively simulating the onset of depression in real life. The environment has certain application value for studying the mechanism of antidepressants and the pathophysiology of depression. This article combines CUMS with the isolation model. In addition to establishing a model quickly and steadily, it can also prevent mice from influencing the structure of each other’s gut microbiota through their feces.

To date, since the etiology of depression is still unclear, the pathogenesis is very complicated, and effective treatment methods are lacking. Generally, the neurotrophin and monoamine hypotheses are recognized. Previous studies demonstrated that a reduction in brain BDNF levels may predict major depressive disorder, while an increase in brain BDNF levels is associated with antidepressant effects (Garcia et al., 2008; Zhou et al., 2014). Depression is associated with decreased levels of monoamine neurotransmitters, such as 5-HT, NE, and DA (Nemeroff, 2007; Racagni and Popoli, 2010; Nagy et al., 2020). Therefore, in addition to behavioral testing, to investigate the potential correlation between the gut microbiota and brain metabolites, we also tested the content of brain-derived neurotrophic factors and monoamine neurotransmitters. In our results, BDNF and monoamine neurotransmitters showed the same trend results as those previously reported, which not only verified the success of the model and demonstrated the effect of alkaloids in improving depression but also laid a better foundation for future research on gut microbes in depressed mice.

The results of our alpha analysis show that the diversity of microbes in the gut of mice treated with drugs (imipramine and alkaloids) decreased. Consistent with the results of previous studies, chronic stress and depression can increase the diversity and richness of the gut microbiota (Naseribafrouei et al., 2014; Jiang et al., 2015; Li et al., 2018), while antibiotics and antidepressants reduce the diversity of microbes in the gut (Manichanh et al., 2010; Lukic et al., 2019). However, some studies have proven that the pressure of CUMS reduces alpha diversity and that antidepressants can improve the reduction in microbial diversity and make the community more stable (Kelly et al., 2016; Bharwani et al., 2017). According to the literature, many factors could affect the diversity of intestinal microbes, such as genetics, environment, age, and sex, which may be the reason for the differences in analysis results (Laukens et al., 2016).

The imbalance of the gut microbiota may be one of the important factors in the development or deterioration of depression. **Figures 4A–E** shows that different levels of microbiota changed under drug treatment. To explore the different microbes in each group, we used LEfSe to analyze the genus level and found that the CUMS group had more differential microbes than the other groups (**Figure 4F**). Among them, the different microbiota comprising the top ten with respect to abundance were *Helicobacter*, *Oscillospira*, *Odoribacter*, *Mucispirillum*, *Ruminococcus*, and *Desulfovibrio*. Previous studies have shown that *Helicobacter pylori* infection results in a significantly higher risk of depressive symptoms (Gu et al., 2019), and the incidence of peptic ulcer disease in patients with depression is twofold higher than that in the general population (Hsu et al., 2015). The relative abundances of *Oscillospira* in the intestines of adrenocorticotropic hormone-induced depression model mice were significantly increased compared with those in the control group, and the abundances were relatively decreased after drug treatment (Song et al., 2019). Compared with that in the control group, the relative abundance of *Odoribacter* in the CUMS group increased significantly (Li N. et al., 2019). Brain BDNF levels were significantly positively correlated with *Odoribacter* (Jiang et al., 2020), and *Mucispirillum* is associated with mood disorders (Getachew and Tizabi, 2019). Some species of *Mucispirillum* are related to intestinal inflammation caused by intestinal “leakage”, and intestinal leakage is also a key factor in the comorbidity of depression and intestinal diseases (Kunugi, 2016; Romijn et al., 2017). *Ruminococcus* is a type of pathogenic bacteria. Studies have reported that traditional Chinese medicine treatments and marketed antidepressants can reduce the abundance of *Ruminococcus* in the intestine (Lukic et al., 2019; Qu et al., 2019). Consistent with the results of a CUMS rat study, the level of *Ruminococcus* in psychotic patients also increased (Clark and Mach, 2016; Zhuang et al., 2018). The *Desulfovibrio* genus produces lipopolysaccharides, and the reduction in *Desulfovibrio* content reduces the release of inflammatory factors, thereby improving depressive behaviors (Zhu et al., 2019). Through the above information on the different microbes in the CUMS group in this study, the gut microbiota and depression are inevitably linked. In addition, our study showed that the *Lactobacillus* genus was differentially abundant in the imipramine treatment group (among the top 10 in overall abundance) and that the relative abundance of *Lactobacillus* also increased significantly after alkaloids treatment. *Lactobacillus* is a well-known beneficial bacterium that also has a positive effect on the central nervous system and plays a role in regulating depression, which has a positive effect on depression. Pressure application leads to a decrease in the content of *Lactobacillus* in the intestine, and oral administration of *Lactobacillus* can improve stress-induced behavior and mild depression (Marin et al., 2017). Moreover, we found that the abundances of these differentially abundant “harmful bacteria” (*Helicobacter*, *Oscillospira*, *Odoribacter*, *Mucispirillum*, *Ruminococcus*, and *Desulfovibrio*) in the gut of CUMS mice were significantly reduced after imipramine and alkaloids treatment (**Supplementary Figure 4**). These changes remind us that one of the mechanisms by which alkaloids exert their antidepressant effects is likely improvement of the gut microbiota, and regulating the microbiota may be a viable treatment for depression.

The bidirectional communication and regulation of the brain and gut microbes play a vital role in depression (Zheng et al., 2016; Yang et al., 2017). When exploring the interaction between the gut microbiota and the brain in CUMS-induced depression in this study, we used RDA to establish the correlation between depression-like behaviors and depression-related indicators and the gut microbiota (top ten differentially abundant microbiota constituents). Excitingly, the results show that there was a certain correlation in general; specifically, the gut microbiota has a strong correlation with SPT, OFT-numbers of crossings, OFT-numbers of rearings, BDNF-h, BDNF-p, 5-HT, and DA. Next, Spearman correlation analyses further showed that the relative abundance of *Lactobacillus*, *Helicobacter*, *Oscillospira*, *Odoribacter*, *Mucispirillum*, and *Ruminococcus* generally appeared to be specifically correlated with depression-like behaviors and depression-related indicators. Previous studies reported that the expression of neurotransmitter (5-HT and DA) receptors is regulated by *Lactobacillus* in the intestine through the vagus nerve (Yu et al., 2017). As discussed earlier, *Helicobacter*, *Oscillospira*, *Odoribacter*, *Mucispirillum*, and *Ruminococcus* were closely related to depression. The prediction results of the gut microbiota metabolism pathway show that there are differences in abundance between the treatment groups. In addition, according to relevant literature reports, research on the combination of gut microbes and metabolomics may make the results of gut microbial metabolic pathways and functions more solid and convincing (Yu et al., 2017; Li J. et al., 2019). From these results, it is not difficult to conclude that gut microbes and related metabolic pathways had a direct or indirect effect on depression symptoms in CUMS mice in our study. *S. alopecuroides* alkaloids have the potential to improve depression by regulating the bidirectional signaling system of gut microbiota and brain.

## Conclusion

In conclusion, the present study shows that alkaloids from *S. alopecuroides* can improve depression in mice and regulate the types of gut microbiota constituents in CUMS mice. The modulation of the relative abundances of key microbiota constituents at the genus level (e.g., *Lactobacillus*, *Helicobacter*, *Oscillospira*, *Odoribacter*, *Mucispirillum*, *Ruminococcus*, *Desulfovibrio*) by alkaloids is beneficial for the improvement of depression. The correlation between gut microbes and depression-like behaviors and depression-related indicators indicates that alkaloids can improve depression in mice by regulating gut microbes. Overall, our research initially revealed the mechanism of action of alkaloids in the treatment of depression, which in turn provides ideas for the study of depression pathogenesis.

## Data Availability Statement

The datasets presented in this study can be found in online repositories. The names of the repository/repositories and accession number(s) can be found below: https://www.ncbi.nlm.nih.gov/, SRP281158.

## Ethics Statement

The animal study was reviewed and approved by the Experimental Animal Care and Use Committee at Northeast Normal University.

## Author Contributions

MZ contributed to the design, data acquisition, analysis, and drafting and critical revision of the manuscript. AL analyzed and wrote the 16S sequencing data. QY, JL, and LW contributed to the data acquisition. XL, YH, and LL designed the study and revised the manuscript. All authors contributed to the article and approved the submitted version.

## Funding

This research was financially supported by the Research Foundation of Jilin Provincial Science & Technology Committee (No. 20190304026YY, 20200404124YYGH, 20200201135JC), the Grant of Ministry of Industry and Information Technology of Changchun City (No. 2017342), the Natural Science Foundation of Jilin Province, P. R. China (No. 20180101242JC), and Systems Biology Research on Genome and Transcriptome of Stem Cells (2017030) of Jilin Province Sunbird Regenerative Medical Engineering Co., Ltd.

## Conflict of Interest

The authors declare that the research was conducted in the absence of any commercial or financial relationships that could be construed as a potential conflict of interest.
