# Supplementary material for: Beneficial Effect of Alkaloids From Sophora alopecuroides L. on CUMS-Induced Depression Model Mice via Modulating Gut Microbiota
Source: Front Cell Infect Microbiol. 2021 Apr 19;11:665159. doi: 10.3389/fcimb.2021.665159 (PMC8089385; doi:10.3389/fcimb.2021.665159)
Supplement: Supplementary file 1 [file DataSheet_1.docx]

Supplementary Material

**Supplementary Tables**

**Supplementary Table 1** Identification of the major alkaloids in *Sophora alopecuroides* L. by HPLC-MS

| Peak (No.) | RT (min) | Predicted formula | Positive ion mode (*m*/*z*) | Identification |
| --- | --- | --- | --- | --- |
| 1 | 23.64 | C_15_H_24_N_2_O_2_ | 265.3 | Oxymatrine |
| 2 | 26.23 | C_15_H_24_N_2_O_2_ | 265.3 | Oxysophoridine |
| 3 | 27.66 | C_15_H_24_N_2_O | 249.4 | Sophoridine |
| 4 | 28.89 | C_15_H_22_N_2_O | 247.3 | Sophocarpine |
| 5 | 30.78 | C_15_H_24_N_2_O | 249.4 | Matrine |
| 6 | 31.66 | C_15_H_24_N_2_O | 249.4 | Lupanine |

**Supplementary Table 2** RDA analysis of the influence of depression-like behaviors and depression-related indicators on the gut microbiota (based on the top ten ASVs).

| Factor | Percent explained | Pseudo-F | P |
| --- | --- | --- | --- |
| SPT | 10.9 | 28.4 | 0.001 |
| FST | 9.6 | 0.1 | 0.859 |
| OFT-numbers of crossings | 21.8 | 4.0 | 0.044 |
| OFT-numbers of rearings | 11.0 | 4.1 | 0.036 |
| OFT-numbers of modifications | -0.7 | 0.5 | 0.461 |
| BDNF-h | 14.7 | 25.0 | 0.001 |
| BDNF-p | 9.4 | 11.9 | 0.003 |
| 5-HT | 11.1 | 5.2 | 0.024 |
| NE | 5.2 | 0.1 | 0.836 |
| DA | 21.0 | 9.0 | 0.005 |
