# Supplementary material for: Beneficial Effect of Alkaloids From Sophora alopecuroides L. on CUMS-Induced Depression Model Mice via Modulating Gut Microbiota
Source: Front Cell Infect Microbiol. 2021 Apr 19;11:665159. doi: 10.3389/fcimb.2021.665159 (PMC8089385; doi:10.3389/fcimb.2021.665159)
Supplement: Supplementary file 2 [file DataSheet_2.pdf]

Supplementary Material

Supplementary Figures

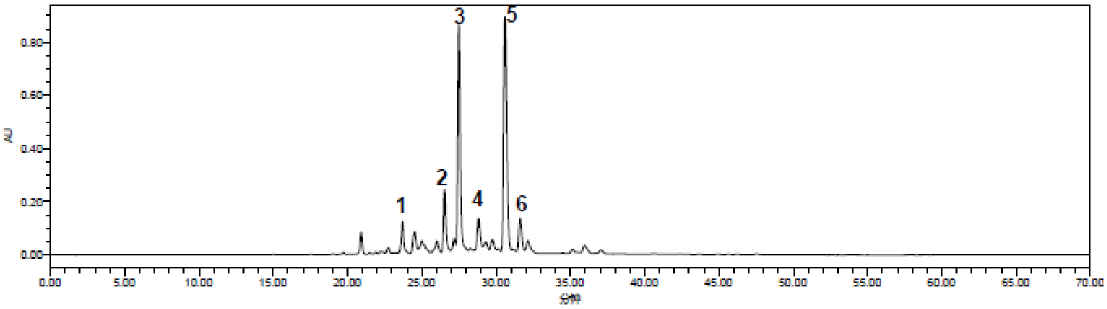

Supplementary Figure 1 The HPLC-MS results of total alkaloids from *Sophora alopecuroides* L..

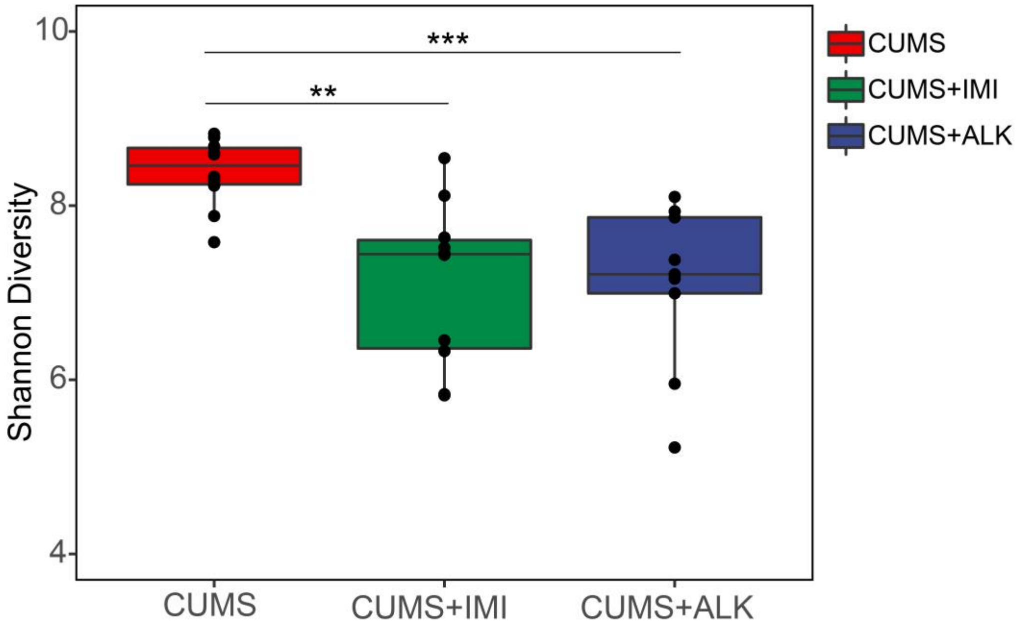

Supplementary Figure 2 The Shannon diversity index of different treatment groups. Data were presented as mean ± SE. Mann-Whitney U test results are shown at the top of each paired comparison. $^{**}P < 0.01$ and $^{***}P < 0.001$ versus the CUMS group.

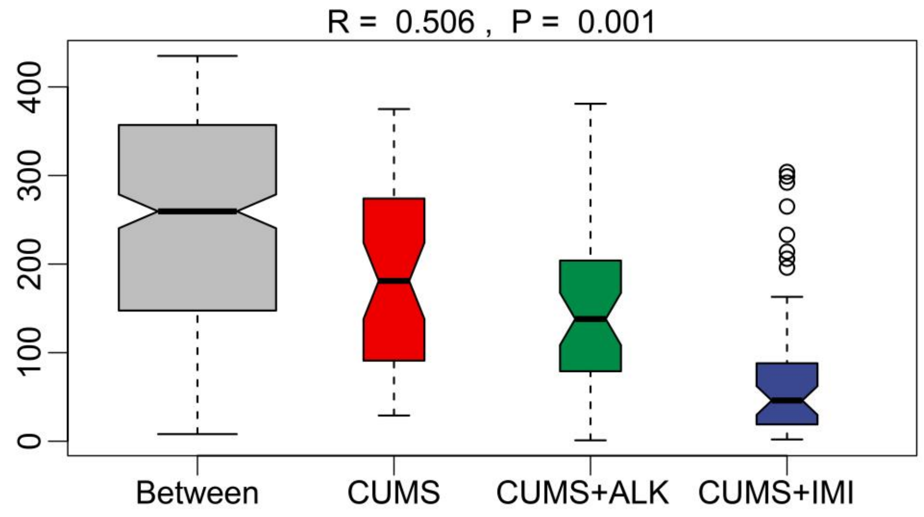

Supplementary Figure 3 The result of ANOSIM analysis based on Bray-Curtis distance.

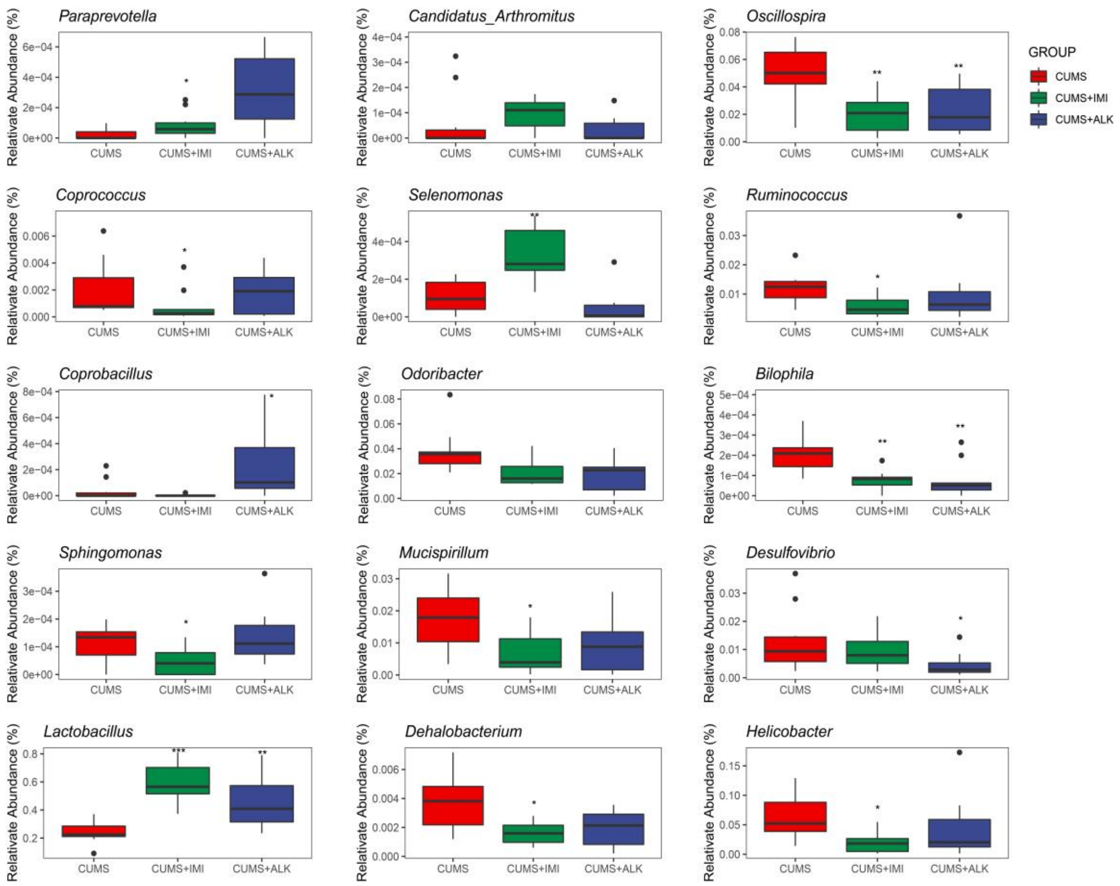

Supplementary Figure 4 Boxplots showing differences in relative abundance of ASVs according to LEfSe analysis (select the top 50 ASVs at the genus level). *$P < 0.05$, **$P < 0.01$, ***$P < 0.001$ versus the CUMS group.

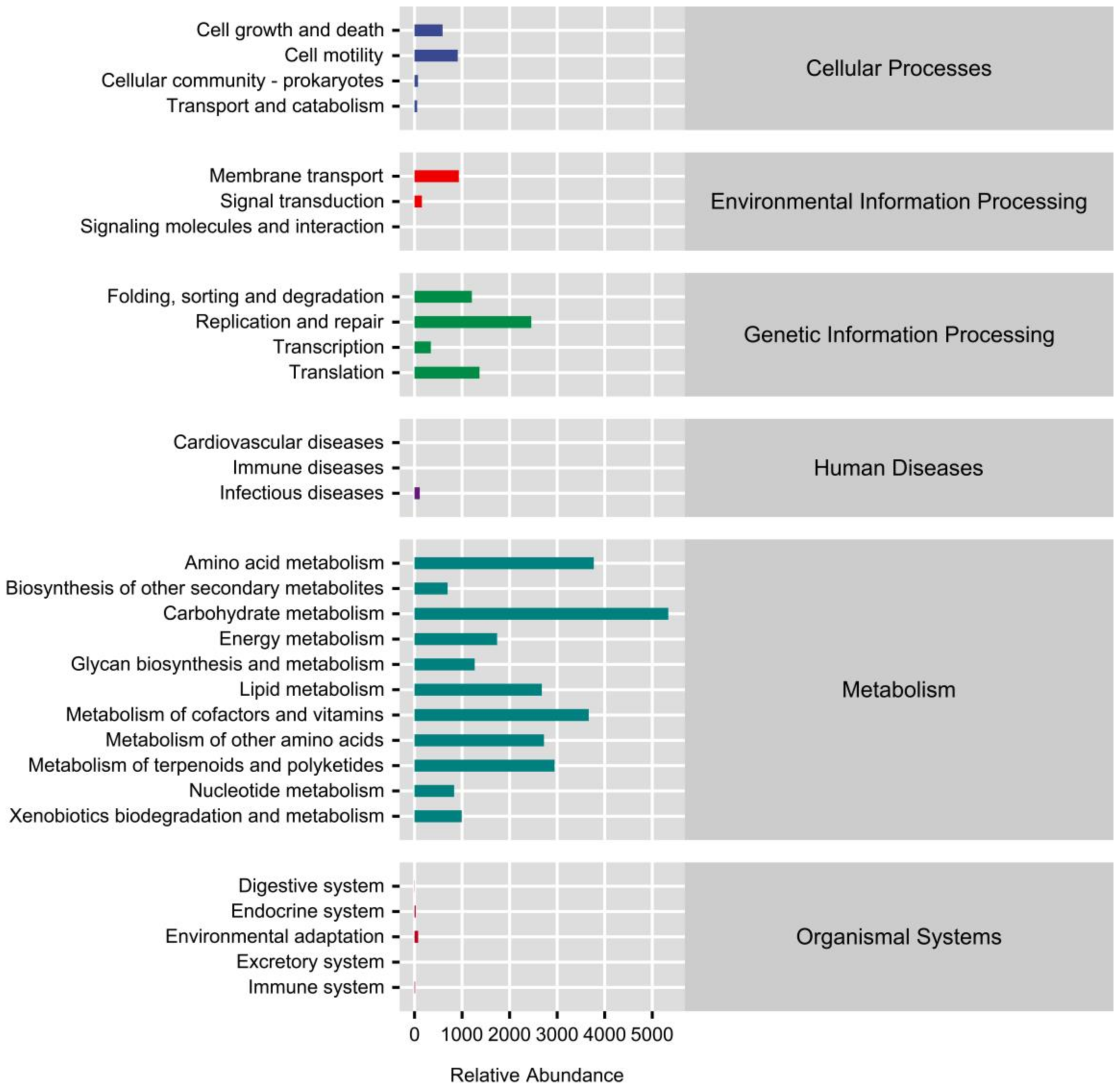

Supplementary Figure 5 Statistical analysis of metabolic pathways in the KEGG Pathway Database. Axis labels: abscissa, relative abundance value; ordinate, different metabolic pathways.
